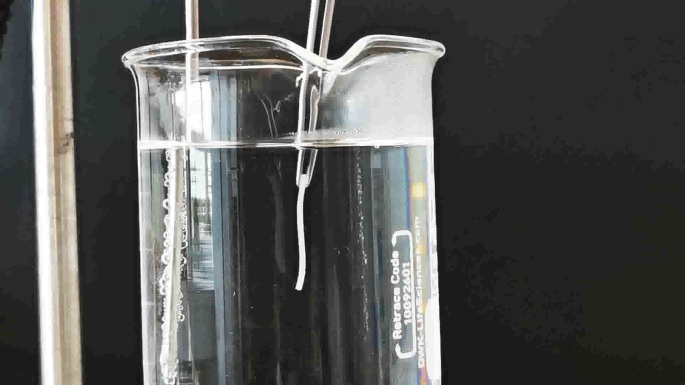# Melt blending of poly(lactic acid) with biomedically relevant polyurethanes to improve mechanical performance

**DOI:** 10.1016/j.heliyon.2024.e26268

**Published:** 2024-02-23

**Authors:** Stefan Oschatz, Selina Schultz, Nicklas Fiedler, Volkmar Senz, Klaus-Peter Schmitz, Niels Grabow, Daniela Koper

**Affiliations:** a Institute for Biomedical Engineering, Rostock University Medical Center, Friedrich-Barnewitz-Straße 4, 18119, Rostock, Germany; b Department Life, Light & Matter (LLM), University of Rostock, 18051, Rostock, Germany; c Institute for ImplantTechnology and Biomaterials e.V., Friedrich-Barnewitz-Straße 4, 18119, Rostock, Warnemünde, Germany

**Keywords:** PLLA, PLLA polyurethane blends, Shape memory polymers, Polymer stent

## Abstract

Minimally invasive surgery procedures are of utmost relevance in clinical practice. However, the associated mechanical stress on the material poses a challenge for new implant developments. In particular PLLA, one of the most widely used polymeric biomaterials, is limited in its application due to its high brittleness and low elasticity. In this context, blending is a conventional method of improving the performance of polymer materials. However, in implant applications and development, material selection is usually limited to the use of medical grade polymers. The focus of this work was to investigate the extent to which blending poly-l-lactide (PLLA) with low contents of a selection of five commercially available medical grade polyurethanes leads to enhanced material properties. The materials obtained by melt blending were characterized in terms of their morphology and thermal properties, and the mechanical performance of the blends was evaluated taking into account physiological conditions. From these data, we found that mixing PLLA with Pellethane 80A is a promising approach to improve the material's performance, particularly for stent applications. It was found that PLLA/Pellethane blend with 10% polyurethane exhibits considerable plastic deformation before fracture, while pure PLLA fractures with almost no deformation. Furthermore, the addition of Pellethane only leads to a moderate reduction in elongation at yield and yield stress. In addition, dynamic mechanical analysis for three different PLLA/Pellethane ratios was performed to investigate thermally induced shape retention and shape recovery of the blends.

## Graphical abstract

**Figure:**
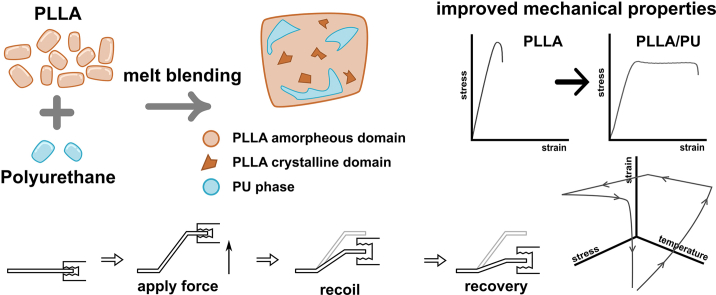

## 1 Introduction

Polymer based bioresorbable stents are regaining interest after the Abbott Absorb shock [1,2], and several new approaches are currently under investigation and in pre- and clinical studies, e.g. Abbott Falcon, Boston Renuvia or MicroPort Firesorb [3]. In this regard, poly(l-lactide) (PLLA) is an established and common used bio- and implant material with a wide range of applications. Due to the minimally invasive implantation strategy, there is a high need for contrary mechanical material specifications in the field of vascular stents. The radial deformability, independent from specific implant design, should ensure both small interventional diameters and deformation reserve for dilating to an appropriate target diameter. Given the primary functional objective of reopening an obstructed vessel, a high radial deformation resistance is required for dilated vascular stents. Material properties such as sufficient plastic deformation capacity (crimping, dilatation), high Young's modulus and yield stress (high radial strength) and structural, as well as mechanical, resistance to cyclic alternating load cases are essential for a functional biomedical device. In particular, mechanical stress during the balloon-assisted dilatation process may lead to fatal implant failure due to fractures, if the material is inadequately ductile. In addition, radial resilience of dilated stents in particular results from plastic deformation behavior.

Yet, its brittleness and low elasticity leave untreated PLLA seemingly less suited for a number of sophisticated biomedical uses where mechanic load occurs, e.g. as material in stent applications. Thus, further processing steps such as an elaborated post processing protocol is required for the manufacture of functional implants, in particular blow molding (Abbott), die drawing (Arterius) or thermal annealing (Elixir) to adjust chain orientation and crystallinity [4,5]. Moreover, to ensure the required mechanical performance, a common approach to adjust polymer mechanical properties is blending with small molecules (as hardener or plasticizer) or with another polymer [6]. In this context, beside using PLLA/PU copolymers [7,8], several PLLA blends and corresponding investigations regarding their thermo-mechanical behavior have been reported in the literature. In particular, it was shown that blending with thermoplastic polyurethanes (TPU) increases elongation at break from ∼5% up to ∼20% and beyond when compared to pure PLLA, paired with distinct plastic deformation, while maintaining material yield strength [9]. The mechanical behavior of PLLA/PU blends, however, depends strongly on the choice of PU, whereas even several 100% elongation at break were observed [[10], [11], [12]]. Yet, phase interaction when blending PLLA with PU is complex, as such blends are also prone to phase separation, with better compatibility when soft segments of TPU, e.g. ether blocks, can interact with PLLA chains [13].

In a biomedical context, however, the use of a material for implant purposes must comply with strict regulatory requirements in terms of biocompatibility and (longterm-) safety, and the materials have to be specifically manufactured for medical purposes. Consequently, polymers designated for implant applications must be assessed regarding the USP classification of plastics, whereas class IV and VI are designated for materials intended for the use in implants and medical devices. In particular, the tests to be conducted according to USP are intravenous and intraperitoneal injection of sample extracts and intramuscular implantation of sample strips [14]. Moreover, tests regarding acute systemic toxicity, intracutaneous reactivity and tissue reaction after implantation have to be performed following DIN-ISO 10993 [15]. These tests should furthermore be in accordance to the Principles of Good Laboratory Practice (GLP). Thus, the choice of blending partners is limited when avoiding or reducing expensive and time-consuming extensive studies for approval, as blending partners should also be of medical grade. In addition, physiological conditions should also be considered during mechanical testing and for evaluating material suitability, as body fluids and corresponding salts can diffuse into the material, interact with the polymer chains, and further affect mechanical performance.

The present work focuses on melt blending medical grade very high molecular weight (VHMW) PLLA, Resomer L210, with small amount of established medical grade polyurethane based polymers. Very and ultra high *M*_*w*_ PLLA is of particular interest for biomedical engineering due to its beneficial properties regarding tissue response and reduced inflammatory potential compared to lower *M*_*w*_ PLLA [16]. The obtained materials were characterized in terms of mechanical and thermal properties, as well as morphology. The following biomedical grade polymers were used as blend partners: Pellethane 2363 80A, and Tecothane TT 1055D, both being aromatic polyether-based thermoplastic polyurethanes, Chronothane P75D, an aromatic polyether-based polyurethane elastomer, Adiamat, a polycarbonate urethane, and ElastEon, a siloxane segmented polyurethane. These polyurethanes have in common that they are proven in biomedical engineering and of major interest in prototyping and manufacturing of biomedical devices and implants, in particular for the manufacture of artificial heart valves [[17], [18], [19]], blood vessel replacement [20] and as insulating material for cardiac pacemaker electrodes [21]. The selected polyurethanes, however, differ in their structural composition and shore hardness. Thus, the influence on mechanical performance and morphology when using these polymers as blending partner with PLLA cannot easily be predicted.

Aim of this study is to obtain a material with a similar tensile strength compared to PLLA paired with enhanced plastic deformation and increased elongation at break. For further evaluating the suitability of the selected PLLA/PU blends as base material for biomedical devices, in particular regarding stent applications, mechanical testing was conducted after incubation in physiological saline solution. Moreover, PLLA/Pellethane blends were selected for further studying their shape memory behavior.

Notably, blending PLLA with polyurethanes seemingly eradicates one of its most beneficial properties regarding biomedical applications – namely biodegradability. Yet, since the polyurethane proportion is low compared to PLLA, what remains is no bulk polymer but only small fragments. Whether these fragments show negative long-term effects on the surrounding tissue must be investigated in further studies, preferably *in vivo*, to assess the advantages of blending with PU over partial loss of degradability.

## 2 Materials and methods

### 2.1 Polymers

All polymers used were of medical grade. PLLA L210 was purchased from Evonik Industries (Essen, Germany), Pellethan 2363 80A was purchased from Lubrizol Advanced Materials (Cleveland, OH, USA), Tecothane TT 1055D was purchased from Velox (Hamburg, Germany), Chronothane P75D was purchased from AdvanSource (Wilmington, MA, USA), Adiamat was purchased from adiam life science AG (Erkelenz, Germany) and ElastEon was provided by RUA life Science (Glasgow, Scotland).

### 2.2 Sample preparation

Blends of PLLA and 10 percentage by weight (wt%) of different PUs were fabricated by melt extrusion using a HAAKE MiniLab II co-rotating twin-screw extruder (Thermo Fisher Scientific, Karlsruhe, Germany) with different screw speeds and nozzle temperatures, listed in Table 2. The extruded filaments were subsequently pelleted. Following this, samples for further testing were fabricated by injection molding using a Haake MiniJet II (Thermo Fisher Scientific, Karlsruhe, Germany) equipped with a 60 × 10 × 1 mm^3^ stainless steel mold. Pure PLLA samples without the addition of PU were fabricated solely by injection molding without previous extrusion process. Extrusion and injection molding parameters are given in Table 2.

**Table 1:** Overview on the polymer structures for PLLA and the Pus used for blend fabrication.

Polymer	Structure	Polymer class	Lit.
PLLA		polyester	
Pellethane		aromatic polyether-based thermoplastic polyurethane	[22]
Tecothane			[23]
Chronothane		aromatic polyether-based polyurethane elastomer^a^	[24]
Adiamat		polycarbonate urethane^b^	[18,25]
ElastEon		siloxane segmented polyurethane	[26]

a General PU elastomer structure. Detailed structural information is not readily available for Chronothane.

b General PCU structure. Detailed structural information is not readily available for Adiamat.

**Table 2:** Extrusion and injection molding parameters for PLLA reference and PLLA/PU blend samples.

Sample name	extrusion			injection molding			
	*T*_*Nozzle*_ (°C)	RPM (1/s)		*T*_*cylinder*_ (°C)	*T*_*mold*_ (°C)	*p*_*injection*_ (bar)	*p*_*hold*_ (bar)
PLLA	/	/		220	67	680 for 5 s	300 for 10 s
PLLA/Pellethane	190	10		205	60	820 for 5 s	300 for 10 s
PLLA/Tecothane	195	10		215	55	600 for 5 s	300 for 10 s
PLLA/Chronothane	185	5		205	55	600 for 5 s	300 for 10 s
PLLA/Adiamat	195	20		225	66	970 for 5 s	300 for 10 s
PLLA/ElastEon	190	20		210	58	750 for 5 s	300 for 10 s

Annealing temperature to diminish cold crystallization has been identified using DSC analysis. Thermal annealing following to the injection molding process was performed using a standard lab heating plate at 85 °C (PLLA) and 75 °C (PLLA/PU blends). Specimens were placed on a common lab heating plate and covered with a previously thermal equilibrated aluminum block equipped with a drilled hole for the heating plate temperature sensor. After thermal annealing for the selected time (see Table 2), samples were cooled under ambient conditions.

### 2.3 Morphology

Macrophotographical imaging was performed using a Canon EOS 80D equipped with a Sigma 18–250 mm macro objective (both Canon Inc., Japan). SEM imaging of cryo-fractured surface morphology was performed with a SEM QUANTA FEG 250 (FEI Deutschland GmbH, Germany) operating in low vacuum with an accelerating voltage of 5 kV, using a secondary electron detector (LVD). Samples were flash-frozen with liquid nitrogen and manually fractured using pincers. To enhance visibility of morphological characteristics, one half of each fractured sample was chemically etched using 1 M NaOH for 72 h, rinsed with distilled water and dried under ambient conditions, whereas the other side of the fracture edge acted as direct reference sample. The specimens were fixed onto aluminum trays with conductive carbon adhesive pads and the aluminum trays were mounted on custom 90° angle brackets to orient fracture edges toward the electron beam source.

### 2.4 Thermal analysis

Thermal properties of the PLLA/PU blends were investigated using a DSC 1 star^e^ system (Mettler Toledo, Zurich, Switzerland). Conventional calibration methods with highly pure standards were used. Test specimens were sliced from injection molded tensile bars and were heated up from *T* = −60 °C at a rate of 10 K/min operating under nitrogen at atmospheric pressure. End temperature was *T* = 240 °C. Sample weights were in the range of *m* = 3–7 mg. Thermograms were analyzed with respect to glass transition (*T*_*g*_), crystallization temperature (*T*_*c*_), melting temperature (*T*_*m*_), and degree of crystallinity (*χ*). Heats of fusion and degree of crystallinity were quantitatively evaluated by determining the endothermic peak areas, which were compared with the melting peak area of totally crystalline PLLA (*χ*_*100*_ = 93.7 J/g) [27]. Degree of crystallinity *χ* for PLLA was quantitatively evaluated by means of Equation (1)

(1) $$\chi =100\%∙\left[\frac{{\Delta H}_{m}}{{\Delta H}_{m0}}\right]∙\frac{1}{W}$$

where Δ*H*_*m0*_ is the enthalpy value of a pure crystalline material, Δ*H*_*m*_ is the enthalpy corresponding to the fusion process and *W* the amount of PLLA component in the PLLA/TPU blend system. All results were averaged over n = 5 samples.

### 2.5 Mechanical analysis

Tensile tests were carried out using a uniaxial tensile test instrument zwickiLine Z 1.0 (Zwick/Roell, Ulm, Germany) equipped with a Canon EOS 80D for simultaneous video recording of sample strain behavior. Samples were cut into 5B dogbone shape specimens according to DIN EN ISO 527-2 [28] using a Speedy 300 CO_2_ laser system (TROTEC Laser GmbH, Marchtrenk, Austria). Measurements were carried out at room temperature under ambient conditions, and in physiological saline solution at an ambient temperature of *T* = 37 °C. For wet measurements, samples were stored for 7 days at 37 °C in physiological saline solution. Tests were conducted with a 1 kN load cell and a crosshead speed of 5 mm/min. Tensile force as a function of elongation was measured. Based on these data, Young's modulus (*E*) was calculated in the linear elastic region. Yield strain (*ε*_*y*_) and yield stress (*σ*_*y*_) were determined using 0.2% offset method, and intersections of the straight lines for *E*_0.2%offset_ with stress-strain curves of the blends were determined using Origin curve intersection minitool (OriginPro 2018b, OriginLab, Northampton, USA). All results were averaged over n = 5 samples.

### 2.6 Shape memory experiments

Shape memory behavior of PLLA and PLLA/Pellethane blends was investigated in line with the method reported by Shirole et al. [29], using a DMA 850 test bench (TA Instruments Inc., USA). Experiments were carried out in single cantilever mode, with a sample size of 17.5 mm × 10 mm x 1 mm. In a first step, the test chamber was heated to strain temperature *T*_*s*_ = 70 °C and the sample was equilibrated for 30 min before a force of 8.0 N was applied at a rate of 0.8 N/min in a second step. The samples were held at 8.0 N for 30 min before temperature was lowered to the fixing temperature *T*_*f*_ = 15 °C at 5 K/min. Samples were kept at *T*_*f*_ for 30 min. Afterwards, the force was released and held at 0 N (force control) for 5 min at 15 °C before the temperature was increased to 70 °C at 5 K/min with *F* set to 0 N. Lastly, samples were kept at 70 °C at *F* = 0 N for 90 min. Starting with the second step, the cycle was repeated twice. From displacement, force and temperature as function of time plots, displacement at strain values *D*_*s*_ were extracted at highest deformation at strain temperature *T*_*s*_, recoil displacement values *D*_*r*_ were extracted as first value after the force was released at fixing temperature *T*_*f*_, and recovery displacement values *D*_*R*_ were extracted as last value at the end of the cycle.

### 2.7 ATR-FTIR spectroscopy

ATR-FTIR-measurements were performed using a Bruker Vertex 70 IR-Spectrometer (Bruker, Leipzig, Germany) equipped with a DLaTGS-detector. Data were collected in the range of $\tilde{\nu }$ = 600 cm^−1^ to 4000 cm^−1^ with a resolution of $\tilde{\nu }$ = 4 cm^−1^ averaged over 32 scans in reflection mode using a Graseby Golden Gate Diamond ATR-unit. All spectra were subsequently baseline corrected and atmospheric compensation has been performed using Bruker OPUS software.

## 3 Results and discussion

For the development of a matching thermal annealing protocol, preliminary tests for each blend were carried out in a DSC device. In an initial step, micro samples were heated to 80 °C (above *T*_*g*_) for selected annealing times (15, 30, 45, 50 and 90 min, respectively). The minimum annealing time, where no more cold crystallization of the material could be observed, was selected and further tests regarding annealing temperature (55, 65 and 75 °C) were conducted for identification of minimal annealing time-temperature pairing, resulting in 85 °C/90 min annealing for pure PLLA, and 75 °C/30 min for all tested PLLA/PU blends. Following this, large-scale samples were thermally annealed using common heating plate and subsequently analyzed by DSC measurements. Thermal annealing parameters for PLLA/PU blends and values for characteristic thermal parameters are given in Table 3.

**Table 3:** DSC results (glass transition (*T*_*g*_), crystallization temperature (*T*_*c*_) and melting temperature (*T*_*m*_)) for PLLA and PLLA/PU blends after injection molding compared to values obtained after injection molding and thermal annealing at given temperature/time.

sample name	as obtained from injection molding				after thermal annealing			
	*T*_*g,PLLA*_ (°C)	*T*_*c,PLLA*_ (°C)	*T*_*m,PLLA*_ (°C)	*χ*_*PLLA*_ (%)	annealing parameters	*T*_*g,PLLA*_ (°C)	*T*_*m,PLLA*_ (°C)	*χ*_*PLLA*_ (%)
PLLA	60.2 ± 0.4	110.1 ± 1.4	179.9 ± 0.4	6.7 ± 1.5	85 °C for 90 min	67.4 ± 0.7	179.9 ± 1.5	48.2 ± 2.6
PLLA/Pellethane	62.0 ± 0.4	83.0 ± 0.4	180.6 ± 0.5	27.2 ± 0.5	all @ 75 °C for 30 min	64.7 ± 0.9	181.0 ± 1.8	64.7 ± 0.9
PLLA/Tecothane	61.0 ± 0.2	88.9 ± 2.8	180.6 ± 1.0	26.0 ± 2.5		63.6 ± 0.8	179.1 ± 1.0	58.4 ± 1.5
PLLA/Chronothane	60.0. ± 0.6	83.6 ± 0.5	180.7 ± 0.9	15.1 ± 1.0		63.9 ± 0.5	177.9 ± 0.5	62.5 ± 0.5
PLLA/Adiamat	62.3 ± 0.4	84.5 ± 0.9	180.1 ± 0.5	8.61 ± 4.4		65.0 ± 0.3	179.3 ± 0.3	51.4 ± 1.2
PLLA/ElastEon	62.5 ± 0.3	83.6 ± 0.5	180.7 ± 0.9	28.6 ± 1.9		63.8 ± 0.8	180.5 ± 0.7	54.5 ± 2.6

Thermograms of PLLA and PLLA/PU blend samples obtained by injection molding before and after thermal annealing are shown in Fig. 1. For reference, corresponding thermograms of pure PU are also given.

**Fig. 1:**
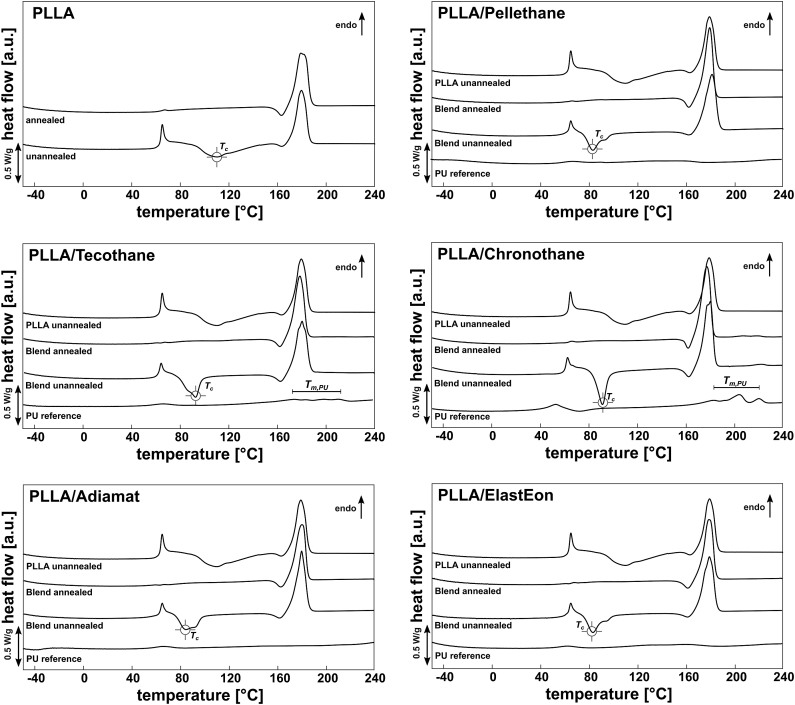
DSC thermograms of PLLA and PLLA/PU-blends as obtained by injection molding and after thermal annealing.

From DSC thermograms, it can be concluded that for pure PLLA and PLLA/PU blends, after injection molding without thermal annealing, the material shows clear cold crystallization signals as well as distinct enthalpy relaxation, indicating that the material is not in equilibrium state due to quenching after sample preparation. Cold crystallization peaks for all PLLA/PU blends were less broad and shifted to lower values compared to pure PLLA. After thermal annealing, no cold crystallization was detected. Blending PLLA with the selected PUs did not show to affect *T*_*m*_, whereas *T*_*g*_ was shifted to lower values after blending.

All Blends showed crystal distortion as indicated by the endothermal peak in front of the melting signal. This is usually an indication of an α’→α transition of PLLA upon heating, but whether the PLLA phase in the blends exhibits such crystals cannot be answered by means of DSC analysis alone. As for pure Tecothane and pure Chronothane, they exhibit a melting range of *T*_*m,Chronothane*_ = 187–221 °C and *T*_*m,Tecothane*_ = 175–219 °C, showing multiple melting peaks, indicating complex crystal structures. When blended with PLLA, only for PLLA/Chronothane a PU melting signal was detected, indicating a separated crystalline PU phase. Pure Adiamat shows a melting temperature peak *T*_*m,Adiamat*_ = 65.8 ± 0.6 °C, overlapping with *T*_*g,PLLA*_, thus, although PU crystallites were found in SEM imaging, no PU melting signal could be detected in the blend. For the other blends, no signs of PU melting were found in DSC thermograms. In general, after thermal annealing, PLLA/PU blends appear to be in a thermal stable condition, which is crucial regarding further processing to biomedical devices and shelf life.

Assuming homogeneous distribution of PUs in the blend in the DSC samples, crystallinity *χ* of PLLA was calculated (Table 3). As obtained by injection molding, pure PLLA is amorphous. After thermal annealing, crystallinity increases to *χ*_*PLLA*_ = 48.2 ± 2.6%. Blending with Pellethane, Tecothane, Chronothane and ElastEon lead to a higher initial crystallinity after injection molding even without thermal annealing, up to *χ*_*PLLA*/ElastEon_ = 28.6 ± 1.9% and *χ*_*PLLA/Pellethane*_ = 27.2 ± 0.5%, which is considerably different from values reported in the literature where no such increase in *χ* was observed for PLLA/PU blends [30]. Also, after thermal annealing, blends with Pellethane, Tecothane, and Chronothane exhibited a higher PLLA-crystallinity of up to *χ*_*PLLA/Pellethane*_ = 64.7 ± 0.9%, although annealing of PLLA/PU blends was performed at a lower annealing temperature, which also corresponds to the lower crystallization temperatures of the PLLA/PU blends. It can be assumed that the higher crystallinity of PLLA in the blends is due to the induced crystallization of PLLA at the PLLA-PU phase boundary [31].

In terms of morphological changes after thermal annealing, macrophotographical images are shown in Fig. 2. Pure PLLA was transparent after injection molding, and turned highly opaque paired with a more rough surface after thermal annealing, indicating recrystallization, also in accordance to DSC thermograms. Except PLLA/Adiamat, all polymer blends were highly opaque after sample preparation without thermal annealing. After thermal annealing, PLLA/Tecothane and PLLA/Chronothane also showed roughening of the surface, and PLLA/Adiamat showed an increase in opacity. Beyond that, no changes in morphology were detected, leading to the assumption that the annealing protocol was both sufficient and low-impact in terms of decomposition.

**Fig. 2:**
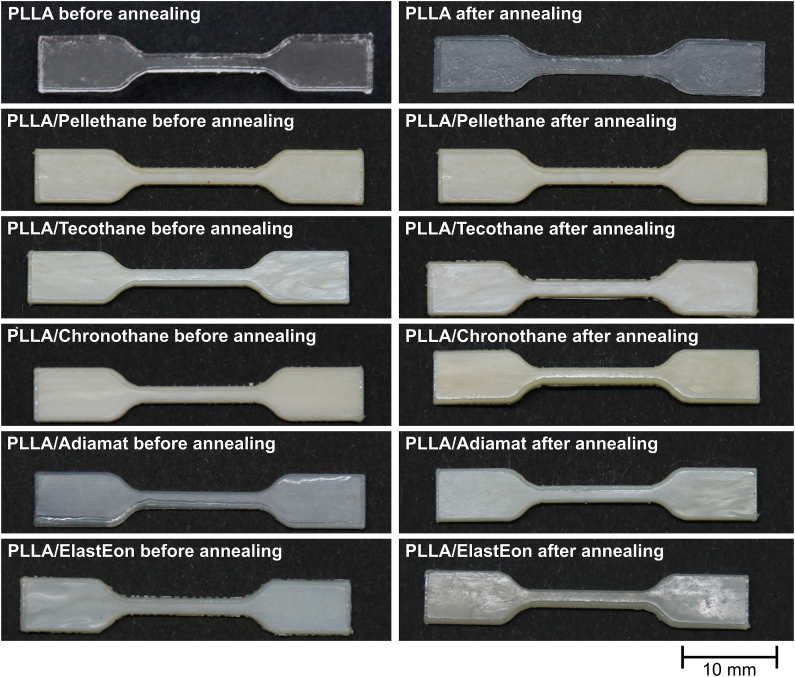
Macrophotographical images of PLLA and PLLA/PU-blend dog bone specimens for mechanical testing as obtained by Laser cutting and after thermal annealing.

To evaluate phase separation and inner morphology, the prepared polymer blends were etched with NaOH (Fig. 3). The basic solution hydrolyzes the amorphous PLLA phase faster than crystalline domains. It is reasonable the selected non-biodegradable PUs to only be slightly affected by hydroxide ions [32]. This effect being due to the butane diol soft segments (Table 1) inhibiting oxidative degradation [33], and the overall hydrophobic nature of PUs furthermore delaying hydrolysis of the urethane groups [34]. Still, a certain degree of depolymerization of PU must be considered. To reduce strain stress on the specimens while cutting, samples were deep frozen using liquid nitrogen and cryo-fractured. One side was not treated with NaOH and served as reference, allowing direct comparison.

**Fig. 3:**
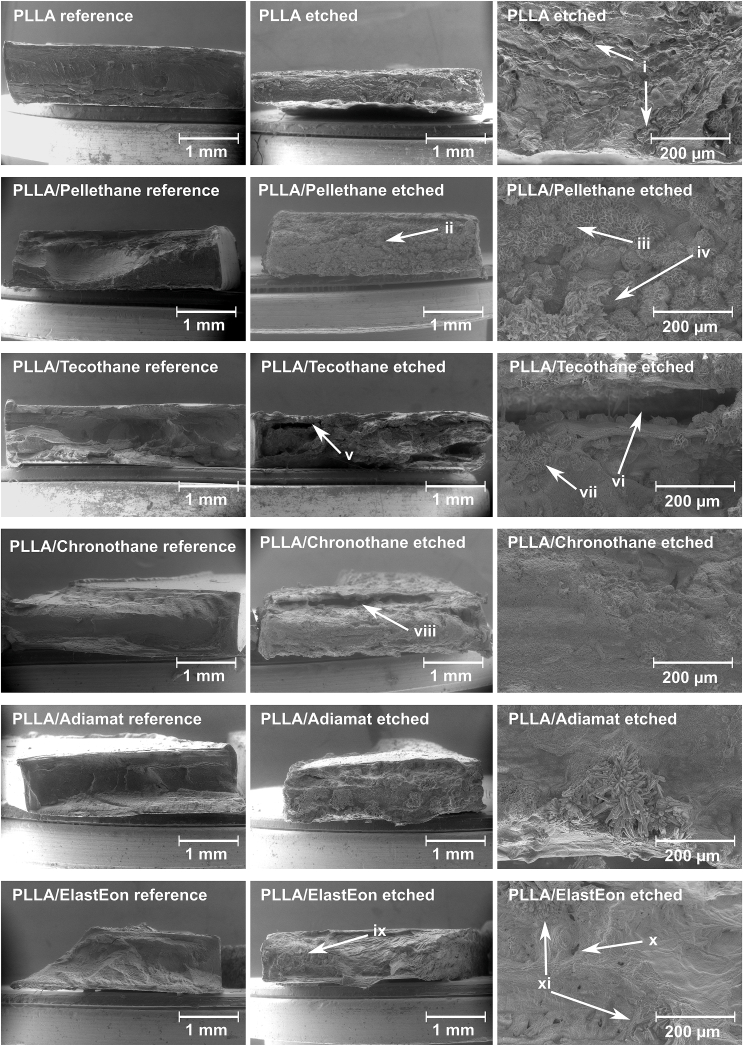
Cross-sectional SEM images of cryo-fractured PLLA/PU blends after etching for 72 h with 1 M NaOH.

Regarding SEM images of the cry-fractured specimens, spherulites of high crystalline domains for pure PLLA became visible after etching (i). For PLLA/Pellethane, a distinguishable PU domain (ii, enlarged at iii and iv) could be observed. As for PLLA/Choronothane (vii), PLLA/Tecothane (v, enlarged at vi) and PLLA/ElastEon (ix, enlarged at x and xi), hollow spaces became visible after etching. These hollows can be attributed to low crystalline PLLA locally surrounded by PU. For PLLA/Adiamat and PLLA/ElastEon (viii), PU crystallites were observed after etching. For the Chronothane blend, however, DSC resulted in multiple melting signals for the polyurethane in the blend at *T*_*m*_ = 187–221 °C. As no large crystallites were found and the *T*_*m*_ signal intensity was very low, we assume Chronothane microcrystallites embedded in the PLLA matrix. For PLLA/Adiamat, no PU melting signal was found, as the melting signal taken from the reference thermogram was overlapping with *T*_*g*_ of PLLA.

**Fig. 4:**
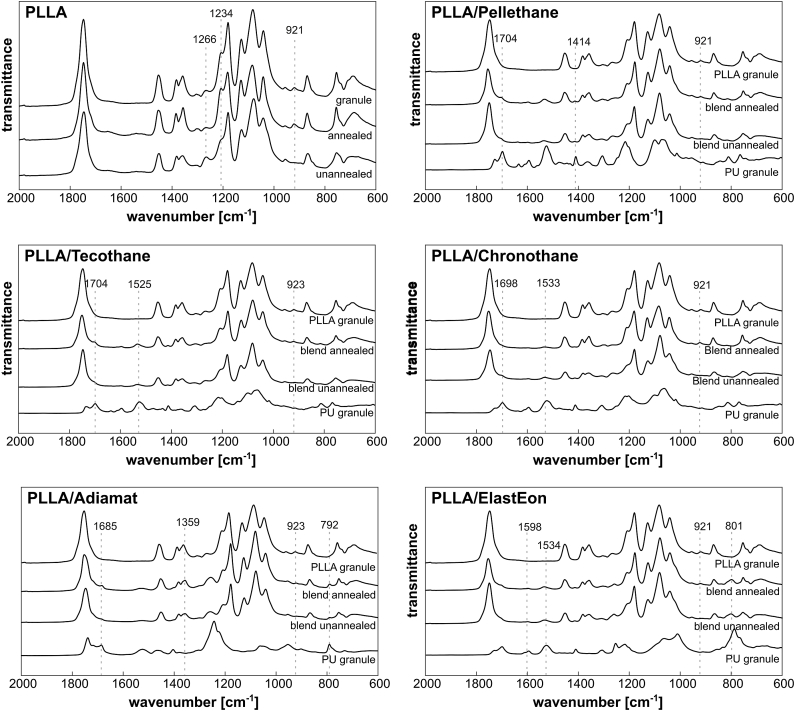
ATR-FTIR spectra of PLLA and PLLA/PU blends before and after thermal annealing. As reference, spectra of pure polymer granules were added. Characteristic bands of PLLA and PU are indicated with dotted lines. To enhance visibility, the spectra were stretched in y-axis.

In the ATR-FTIR spectrum (Fig. 4), PLLA shows characteristic changes in crystallinity sensitive bands after thermal annealing, in particular at $\tilde{\nu }$ = 921 cm^−1^, but also at $\tilde{\nu }$ = 1234 cm^−1^ and $\tilde{\nu }$ = 1266 cm^−1^, indicating an increase in crystallinity [35,36]. The changes at $\tilde{\nu }$ = 921 cm^−1^ band are also visible for all PLLA/PU blends, except PLLA/Adiamat, where this band is overlaid with PU bands. In addition, the respective PU characteristic bands were observable after blending. As no distinct changes in bands for the blends attributed to PLLA were detectable, it can be concluded that there is no interaction between the respective polymers on a molecular level when being blended.

**Fig. 5:**
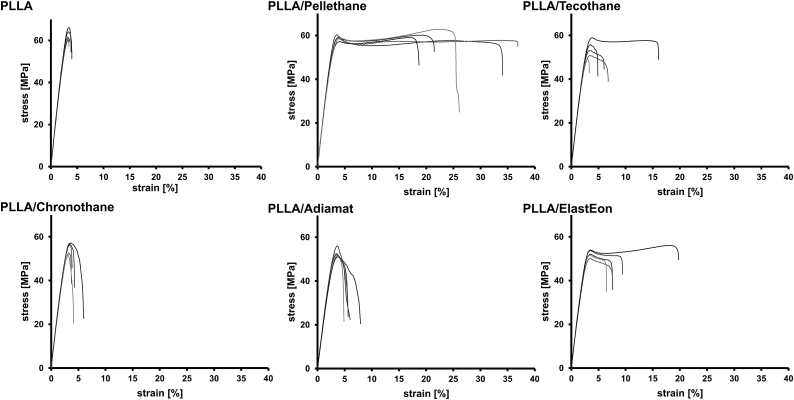
Stress strain curves of PLLA and PLLA/PU blends after injection molding and thermal annealing measured in dry state.

To evaluate the extent to which blending with 10 wt% biomedical grade PU leads to an improvement in elasticity and plastic deformability, uniaxial tensile tests have been performed of the dry blends (Fig. 5). Pure PLLA obtained by injection molding showed a strain at break of *ε*_*B*_ = 3.6 ± 0.2%, which is comparable to results reported in the literature [37]. Blending with 10 wt% Pellethane lead to an increase in *ε*_*B*_, paired with distinct plastic deformation before break. Tecothane as blending partner showed the tendency for only a slight increase in *ε*_*B*_. Blending with Chronothane and Adiamat did not lead to such an increase in *ε*_*B*_ and the blends did not show distinct plastic deformation, which can be attributed to the PU crystals, which were found in cross-sectional SEM images. Blending with ElastEon lead to comparable results as with Pellethane. However, blending with Pellethane, Tecothane and ElastEon lead to an increase in statistical variance.

For the suitability of a polymer for biomedical devices, mechanical performance is a crucial aspect. PLLA in particular, although exhibiting high tensile strength, is limited in its uses due to its brittleness and low plasticity. The increase in *ε*_*B*_ can be attributed to the PU phase, providing sufficient ductility. Where PLLA due to its rigid nature and brittleness easily breaks, embedding PUs may allow for a considerably higher strain, increasing *ε*_*B*_ by 660% in the case of Pellethane ($\bar{\varepsilon }$_*B,PLLA/Pellethane*_ = 28 ± 7% vs. $\bar{\varepsilon }$_*B*_,_*PLLA*_ = 3.6 ± 0.3%, measured in dry state, Fig. 5) as blending partner.

Notably, both, Pellethane and Tecothane are aromatic polyether-based thermoplastic polyurethanes with a comparable chemical composition. However, stress strain-curves revealed a highly different influence on strain at break when blended with PLLA ($\bar{\varepsilon }$_*B,PLLA/Pellethane*_ = 28 ± 7% versus $\bar{\varepsilon }$_*B,PLLA/Tecothane*_ = 7 ± 5%). We conclude that this is due to the different content of soft segment in the PU, which is directly indicated by the difference in shore hardness of 80A (Pellethane) versus 55D (Tecothane). The soft segment can in turn interact with PLLA phase, thus, the higher the soft segment, the softer the materials (Pellethane) and the higher the influence on *ε*_*B*_*.* However, it was shown that the molecular differences of all PUs (soft segment content, siloxane content and carbonate content) as blending partners did not show to have a different influence on Young's modulus and yield point (Table 4).

**Table 4:** Young's modulus values, yield strain (*ε*_*y*_) and yield stress (*σ*_*y*_) for PLLA/PU blends with 10 wt% PU after injection molding and thermal annealing measured in dry state and after incubation in physiological saline solution for 7 days, extracted from stress-strain curves.

	PLLA	PLLA/Pellethane	PLLA/Tecothane	PLLA/Chronothane	PLLA/Adiamat	PLLA/ElastEon
*E* dry (MPa)	2370 ± 50	2227 ± 50	2145 ± 83	2070 ± 60	1930 ± 260	2101 ± 23
*E* medium (MPa)	2280 ± 40	1920 ± 70	1915 ± 79	2090 ± 40	1800 ± 100	1942 ± 78
*ε*_*y*_ dry (%)	2.56 ± 0.11	2.27 ± 0.04	2.26 ± 0.12	2.55 ± 0.19	2.58 ± 0.06	2.25 ± 0.14
*ε*_*y*_ medium (%)	2.62 ± 0.13	2.44 ± 0.18	2.47 ± 0.12	2.50 ± 0.18	2.56 ± 0.15	2.47 ± 0.14
*σ*_*y*_ dry (MPa)	55.7 ± 2.3	46.0 ± 1.5	44.0 ± 1.1	49 ± 4	46.0 ± 1.6	43 ± 3
*σ*_*y*_ medium (MPa)	55 ± 4	43 ± 5	43.5 ± 2.0	48 ± 5	43 ± 4	44.1 ± 1.4

**Fig. 6:**
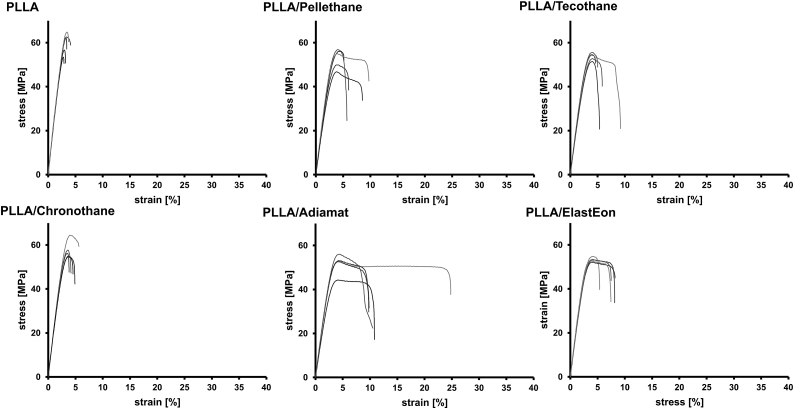
Stress strain curves (averaged over 5 measurements) of PLLA and PLLA/PU blends after injection molding and thermal annealing measured after incubation in physiological saline solution for 7 d.

To further investigate on how the PLLA/PU blends perform under relevant conditions in terms of implant application, polymer specimens were incubated for 7 days in physiological saline solution and stress strain curves were measured from wet samples (Fig. 6). Investigating mechanical performance in medium contact must be considered for biomedical applications, as water and ions from the medium diffuse into the polymer and may affect mechanical properties.

After storing in medium, for pure PLLA, PLLA/Tecothane, PLLA/Chronothane and PLLA/ElastEon, no pronouned changes in mechanical behavior were observed compared to dry state. For PLLA/Pellethane and PLLA/Adiamat, a decrease in *ε*_*B*_ was found. Notably, for PLLA/Pellethane stress strain curves showed less variance for the wet tested specimens compared to the dry ones. In the case of PLLA/Adiamat blend, after incubation, the material showed an increase in *ε*_*B*_ paired with plastic deformation, which could not be observed for the dry tested samples. Plastic deformation, as observed for PLLA/Pellethane, PLLA/Tecothane, PLLA/ElastEon and, when wet, for PLLA/Adiamat, is a crucial parameter for biomedical uses, in particular regarding application as stent material, as crimping and dilation require the material must withstand these processes without failure.

**Fig. 7:**
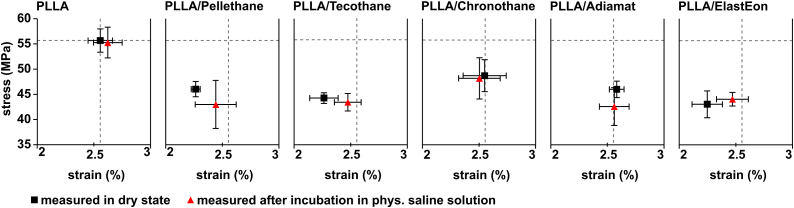
Yield stress versus yield strain of PLLA/PU blends with 10 wt% PU after injection molding and thermal annealing measured in dry state and after incubation in physiological saline solution for 7 days, calculated from stress-strain curves by 0.2% offset method. Dashed line indicate PLLA values, measured in dry state as reference.

From stress strain curves of dry and wet tested blends, the material parameters yield strain (*ε*_*y*_) and yield stress (*σ*_*y*_) were extracted to evaluate the plastic resilience of the materials (Table 4 and Fig. 7). Incubation in physiological saline has no effect on the *E* value of PLLA, but decreased *E* in the case of blending with Pellethane, Tecothane and ElastEon.

Overall, blending did lead to a decrease of *ε*_*y*_ and *σ*_*y*_ compared to pure PLLA. Still, all materials showed to endure a strain of minimum 2.2% without plastic deformation, as can be seen from overview of yield stress versus yield strain in Table 4 and Fig. 7. The values remained comparable for each blend either in dry or wet state. As for the yield stress, the value decreased for all the blends with 10 wt% Polyurethane, with the lowest value dropping from *σ*_*y*_ = 55.67 ± 2.3 MPa for pure PLLA to *σ*_*y*_ = 43.0 ± 3 MPa for PLLA/Adiamat. This in turn may affect the performance of the material, in particular for stent application, where radial fatigue is a critical parameter. However, how the differences in yield strength affect the resulting radial force, including the influence of other parameters such as implant design, needs to be evaluated for specific stent prototypes.

**Fig. 8:**
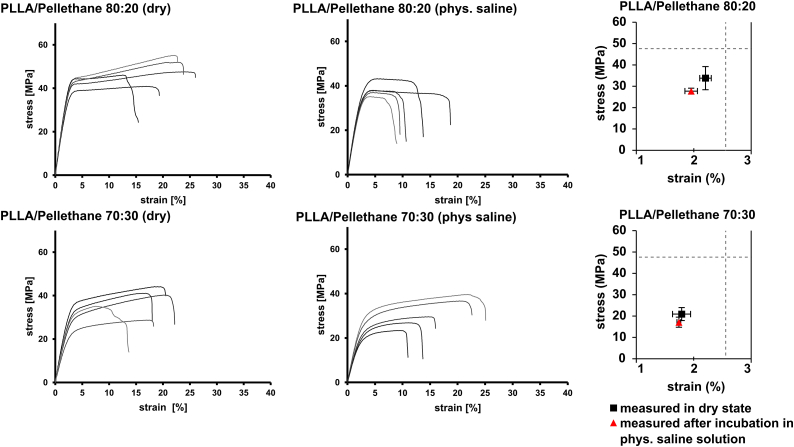
Stress strain curves of PLLA/Pellethane blends with a ratio of 80:20 and 70:30 after injection molding and thermal annealing measured in dry state, and yield stress versus yield strain of PLLA/Pellethane 80:20 and 70:30 measured in dry state and after incubation in physiological saline solution for 7 days, calculated from stress-strain curves by 0.2% offset method. Dashed line indicate PLLA values measured in dry state as reference.

To further evaluate the effect of PU as blending partners on mechanical properties, PLLA/Pellethane blends with higher PU fractions, 20 and 30 wt% Pellethane in the blend, were investigated (Fig. 8).

First, blending 20 wt% of Pellethane lead to a further increase in *ε*_*B*_, as well as reduction of scattering of the resulting values for PLLA/Pellethane 80:20, indicating a higher homogeneity of the blend. A higher amount of 30 wt% Pellethane did not show to further affect *ε*_*B*_. However, blending with higher amounts of Pellethane lead to a further decrease of *ε*_*y*_ and *σ*_*y*_, down to *σ*_*y,70:30*_ = 20.88 ± 3.05 MPa and *ε*_*y,70:30*_ = 1.79 ± 0.16% (Fig. 8 and Table 5).

**Table 5:** Young's modulus values, yield strain (*ε*_*y*_) and yield stress (*σ*_*y*_) for PLLA/Pellethane 80:20 and 70:30 measured in dry state and after incubation in physiological saline solution for 7 days, extracted from stress-strain curves.

	PLLA/Pellethane 80:20	PLLA/Pellethane 70:30
*E* dry (MPa)	1674 ± 206	1336 ± 112
*E* medium (MPa)	1591 ± 79	1110 ± 131
*ε*_*y*_ dry (%)	2.21 ± 0.10	1.79 ± 0.16
*σ*_*y*_ dry (MPa)	34 ± 6	21 ± 3
*ε*_*y*_ medium (%)	1.95 ± 0.11	1.74 ± 0.03
*σ*_*y*_ medium (MPa)	27.8 ± 1.4	17.2 ± 2.3

**Fig. 9:**
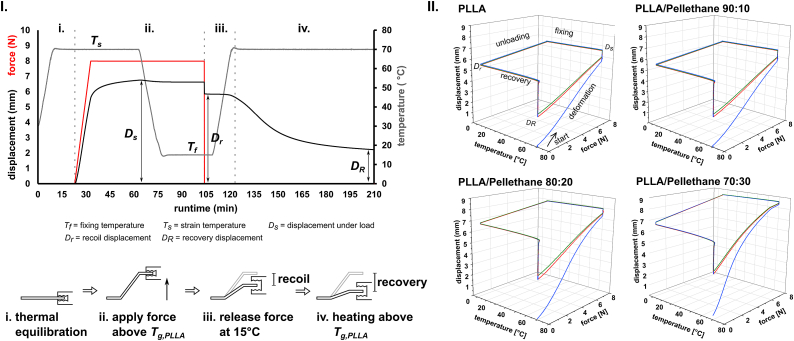
I: Representative diagram of displacement, force and temperature as function of time for pure PLLA for the first complete DMA cycle for SM characterization and schematic of the mechanical load on the sample. Characteristic values *D*_*s*_, *D*_*r*_ and *D*_*R*_ are indicated. 2nd and 3rd cycles were similar, except the initial thermal equilibration step (i) was skipped. DMA protocol was the same for all measured samples. II: Representative 3D plots of DMA measurements for PLLA and PLLA/Pellethane blends (90:10, 80:20 and 70:30) after three cycles. Colors are indicative for cycle 1 (blue), cycle 2 (red) and cycle 3 (green). Thermal equilibration step is not shown in 3D plots.

**Fig. 10:**
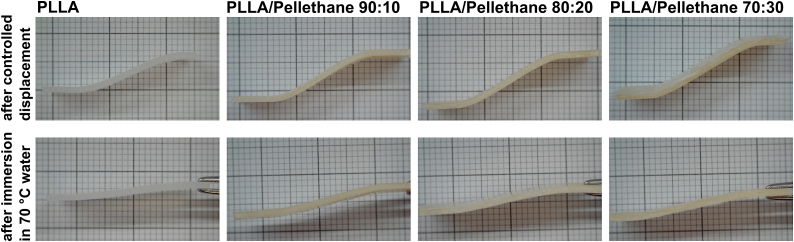
Photographic images of PLLA and PLLA/Pellethane after controlled displacement using DMA device. The test chamber was heated to 70 °C and the sample was equilibrated for 30 min before a force of 8.0 N was applied at a rate of 0.8 N/min. The samples were held at 8.0 N for 30 min before temperature was lowered to 15 °C. Samples were kept at 15 °C for 30 min, and then the force was released. The bottom line shows photographic images of the same samples after 1 min immersion in deionized water heated to 70 °C.

PLLA/polyurethane blends have gained attention in implant development, as they have been reported to show thermally induced shape memory (SM) behavior [31,38]. In brief, the amorphous domain acts as reversible phase, enabling shape recovery when heated above *T*_*g*_ [39]. As for PLLA and PLLA/PU blends, SM results from the deformation of the polymer above *T*_*g*_ of PLLA, providing sufficient plasticity as the amorphous regions are in the viscous state. PLLA crystallites then serve as anchor points for highly flexible phase. According to the literature, in the case of PLLA/PU blends, the rubbery elastic PU phase in addition acts as load carrier, preventing PLLA from breaking and allowing higher elongation. On cooling under load, the energy is stored in the material. When polymer is then reheated above *T*_*g,PLLA*_, the material regains molecular mobility, causing recovery to the initial shape [31]. SM polymers are of high interest in applications such as 4D printing [40], but also as material in biomedical engineering. Based on the mechanical data for all blends, SM behavior of PLLA/Pellethane was selected to be further investigated for 90:10, 80:20 and 70:30 blends using DMA (Fig. 9, Fig. 10, Fig. 10 and Table 6).

**Table 6:** Shape memory parameters displacement under load (*D*_*s*_), recoil displacement (*D*_*r*_) and recovery displacement (*D*_*R*_) for the first and the third cycle obtained by DMA experiments as shown in Fig. 9.

	first cycle (n = 3)			third cycle (n = 3)		
	*D*_*s*_ (mm)	*D*_*r*_ (mm)	*D*_*R*_ (mm)	*D*_*s*_ (mm)	*D*_*r*_ (mm)	*D*_*R*_ (mm)
PLLA	6.5 ± 0.3	5.7 ± 0.3	2.4 ± 0.2	6.6 ± 0.5	5.7 ± 0.4	2.8 ± 0.2
PLLA/Pellethane 90:10	8.1 ± 0.1	7.2 ± 0.1	3.3 ± 0.1	8.1 ± 0.1	7.2 ± 0.1	3.7 ± 0.1
PLLA/Pellethane 80:20	8.1 ± 0.3	7.2 ± 0.2	3.5 ± 0.1	8.1 ± 0.2	7.1 ± 0.2	3.9 ± 0.1
PLLA/Pellethane 70:30	8.5 ± 0.1	7.1 ± 0.1	3.9 ± 0.9	8.5 ± 0.1	7.1 ± 0.1	4.2 ± 0.1

In DMA experiments (Fig. 9 I), PLLA and PLLA/Pellethane blends showed a recoil after fixation at 15 °C and releasing the force (*D*_*s*_-*D*_*r*_). When the heating above *T*_*g*_, all of the selected polymers exhibited recovery towards the initial state (*D*_*R*_). The recovered state can then be restored in cyclic experiments (Fig. 9 II). From the DMA experiments, it can be seen that blending with PU leads to higher displacement under load due to the increased flexibility of the material, also in accordance with uniaxial tensile tests. In addition, with increasing PU fraction, recovery decreases (Table 7).

**Table 7:** Shape retention rate = *D*_*r*_x100/*D*_*s*_ and shape recovery rate = (*D*_*r*_-*D*_*R*_)x100/*D*_*s*_ calculated from SM parameters (Table 6) according to Lu et al. [41].

	Shape retention rate for the first cycle (n = 3)	Shape recovery rate for the first cycle (n = 3)	Shape retention rate for the third cycle (n = 3)	Shape recovery rate for the third cycle (n = 3)
PLLA	87.2 ± 0.2%	73.2 ± 3.2%	86.6 ± 0.5%	66.6 ± 3.2%
PLLA/Pellethane 90:10	88.4 ± 0.3%	66.9 ± 0.5%	88.3 ± 0.3%	61.6 ± 0.7%
PLLA/Pellethane 80:20	88.2 ± 0.7%	64.3 ± 2.3%	87.9 ± 0.7%	58.6 ± 2.9%
PLLA/Pellethane 70:30	83.6 ± 0.5%	65.5 ± 1.3%	83.1 ± 0.6%	61.5 ± 1.7%

For better visualization, shape recovery was additionally investigated on samples that were displaced in a controlled manner using the DMA device and thermal shape recovery was induced by immersion in water at 70 °C.

Videos of the thermally induced shape recovery by immersion in 70 °C water have been recorded and are provided below:

Supplementary video related to this article can be found at https://doi.org/10.1016/j.envres.2024.118137

The following are the supplementary data related to this article:

Video 1 Thermally in-duced shape recovery of PLLA/PE 100:0. Video 1

Video 2 Thermally in-duced shape recovery of PLLA/PE 90:10. Video 2

Video 3 Thermally in-duced shape recovery of PLLA/PE 80:20. Video 3

Video 4 Thermally in-duced shape recovery of PLLA/PE 70:30. Video 4

Shape retention and thermally induced shape recovery of PLLA/Pellethane blends were determined using DMA. Our finding was that SM behavior is not as pronounced for the selected PLLA/Pellethane blends as for pure PLLA. However, when considered in conjunction with the mechanical performance, in particular the higher plasticity, the blends may be of benefit regarding the material performance for stent applications, manufacturing and processing. Notably, SM behavior of PLLA and PLLA/Pellethane is triggered when heated above *T*_*g,PLLA*_ = ∼68 °C. As this is above body temperature, SM is not of particular relevance for direct exploitation when implanted. However, it may be highly beneficial for manufacturing of biomedical devices, in particular in the context of polymer stents, as the transition of a temporary to the initial shape may facilitate stent dilation in the body. Although SM is not as pronounced in PLLA blends compared to SM alloys such as nitinol, it may help overcome one of the main disadvantages of PLLA, its brittleness and low plasticity, and enable innovative device designs.

## 4 Conclusion

Blending PLLA with polyurethanes is a powerful tool to improve the material's mechanical performance for biomedical applications, particularly in the context of biodegradable polymer stent development. In this context, thermal and mechanical properties are of major importance to be able to develop functional and safe biomedical devices. However, the choice of blending partners is limited to medical grade materials to bypass time-consuming and expensive approval processes. The chemical structure of the polyurethane influences the interaction of the polymers when blended, leading to complex phase behavior. Our finding was, that from the selected polyurethanes, in particular blending PLLA with Pellethane lead to a major improvement of the mechanical characteristics paired with thermal stability and good shape recovery properties, which, in turn, has the potential to open for new aspects in stent manufacturing processes.

## Data availability statement

The raw/processed data required to reproduce these findings cannot be shared at this time due to technical or time limitations.

## CRediT authorship contribution statement

**Stefan Oschatz:** Writing – original draft, Visualization, Methodology, Data curation, Conceptualization. **Selina Schultz:** Writing – review & editing, Methodology, Data curation. **Nicklas Fiedler:** Writing – original draft, Methodology. **Volkmar Senz:** Writing – review & editing, Methodology. **Klaus-Peter Schmitz:** Writing – review & editing, Funding acquisition. **Niels Grabow:** Writing – review & editing, Funding acquisition. **Daniela Koper:** Writing – original draft, Methodology, Investigation, Conceptualization.

## Declaration of competing interest

The authors declare that they have no known competing financial interests or personal relationships that could have appeared to influence the work reported in this paper.

## Figures and Tables

**figs1:**
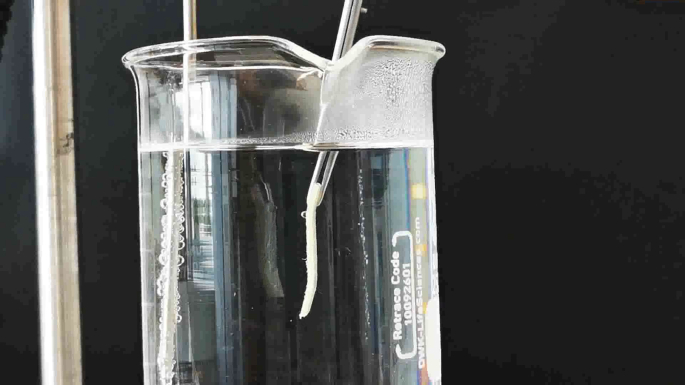


**figs2:**
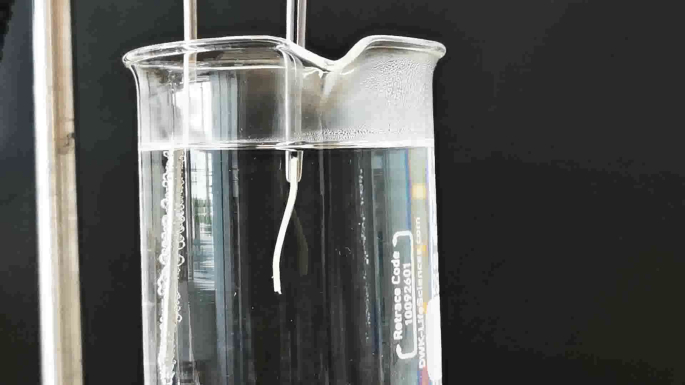


**figs3:**
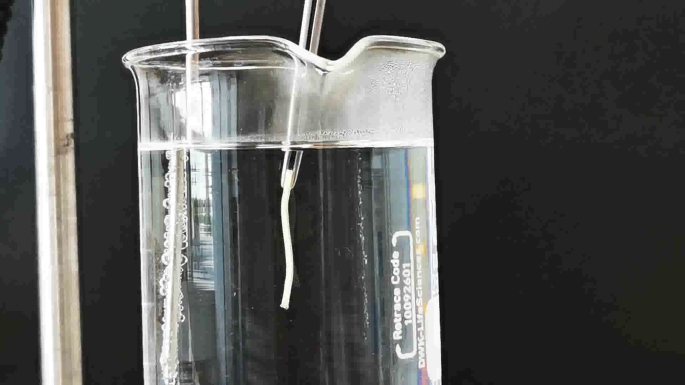


**figs4:**